# Chronic treatment with the (iso-)glutaminyl cyclase inhibitor PQ529 is a novel and effective approach for glomerulonephritis in chronic kidney disease

**DOI:** 10.1007/s00210-020-02013-x

**Published:** 2021-03-29

**Authors:** Naotoshi Kanemitsu, Fumiko Kiyonaga, Kazuhiko Mizukami, Kyoichi Maeno, Takashi Nishikubo, Hiroyuki Yoshida, Hiroyuki Ito

**Affiliations:** 1 Development, Astellas Pharma Inc., 2-5-1, Nihonbashi-Honcho, Chuo-ku, Tokyo, 103-8411 Japan; 2 Corporate Advocacy, Astellas Pharma Inc., Chuo-ku, Tokyo, 103-8411 Japan; 3 Drug Discovery Research, Astellas Pharma Inc., Tsukuba-shi, Ibaraki 305-8585 Japan; 4 Astellas Innovation Management LLC, 1030 Massachusetts Ave. Suite 310, Cambridge, MA 02138 USA

**Keywords:** QC/isoQC inhibitor, PQ529, CCL2/CCR2 axis, Glomerulonephritis, Chronic kidney disease

## Abstract

Glomeruli and renal tubule injury in chronic kidney disease (CKD) is reported to involve induction of macrophage activation through the CCL2/CCR2 axis. The effects of inhibitors of the CCL2/CCR2 axis, such as anti-CCL2 antibody and CCR2 antagonist, on kidney function in animal models or humans with kidney dysfunction have been demonstrated. The N-terminal glutamine on immature CCL2 is replaced with pyroglutamate (pE) by glutaminyl cyclase (QC) and isoQC. pE-CCL2 is stable and resistant to peptidases. We hypothesized that inhibiting QC/isoQC activity would lead to the degradation of CCL2, thereby ameliorating CKD and reducing kidney inflammation. To test this hypothesis, we investigated the renoprotective properties of the QC/isoQC inhibitor PQ529 in anti-glomerular basement membrane (GBM) antibody–induced glomerulonephritis Wistar Kyoto (WKY) rats. Three-week repeated administration of PQ529 (30 and 100 mg/kg, twice daily) significantly reduced the serum and urine CCL2 and urinary protein excretion in a dose-dependent manner. Correlations between the urinary protein level and serum or urinary CCL2 levels were confirmed in tested animals. Repeated administration of PQ529 significantly reduced the expression of CD68, a macrophage marker, in the kidney cortex and mononuclear infiltration into the tubulointerstitium. In addition, decreased levels of urinary KIM-1, β2 microglobulin, and clusterin were detected, suggesting the inhibition of inflammation in both the proximal and distal tubules. These results suggest that PQ529 suppresses the progression of inflammation-induced renal dysfunction by inhibiting the CCL2/CCR2 axis. Inhibition of QC/isoQC may thus be a viable alternative therapeutic approach for treating glomerulonephritis and CKD patients.

**Supplementary Information:**

The online version contains supplementary material available at 10.1007/s00210-020-02013-x.

## Introduction

Chronic kidney disease (CKD) is a condition characterized by the poor long-term function of the kidneys. CKD is reportedly quite prevalent, and over 10% of the adult population in developed countries is estimated to have some degree of CKD (Lopez-Novoa et al. 2010). CKD is usually caused by other conditions that place strain on the kidneys, often resulting from a combination of various problems, such as high blood pressure, diabetes, high cholesterol, kidney infection, and glomerulonephritis as well-known causes. In the general population, a decreased renal function is considered an independent risk factor for both cardiovascular disease and all-cause mortality.

However, while the causes are well known, a cure for CKD remains elusive. Although a number of treatments have been developed, such as angiotensin-converting enzyme (ACE) inhibitors and angiotensin receptor blockers (ARBs) (Berl 2009; Macconi 2010), the absolute risk of renal and cardiovascular morbidity and mortality in CKD patients remains high (Lambers Heerspink and de Zeeuw 2013). Therefore, novel CKD treatments remain necessary as mono- or add-on therapy to these ACE inhibitors and ARBs.

CCL2 (C-C motif ligand 2, MCP-1) is a small cytokine classified as a member of the CC chemokine family. CCL2 exerts a chemoattractive activity toward monocytes, macrophages, and other immune cells that express CCR2 (C-C chemokine receptor type 2, CD192) on their cell surface, inducing the migration of these cells to inflammatory sites (Carr et al. 1994; Xu et al. 1996; Gschwandtner et al. 2019). Several reports have shown that the activation of macrophage induction through the CCL2/CCR2 axis particularly induces injury to the nephron and renal tubes in CKDs, such as glomerulonephritis and diabetic kidney diseases (Wada et al. 2000; Tesch et al. 1999; Giunti et al. 2010; Tampe and Zeisberg 2014; Haller et al. 2016; Moreno et al. 2018). Under these conditions, CCL2 is overexpressed in the tubulointerstitium and glomerulus (Viedt et al. 2002). Macrophages migrate to and damage these sites, leading to glomerulonephritis and the leakage of proteins into the urine (Banba et al. 2000; Eardley et al. 2006).

The effects of inhibiting the CCL2/CCR2 axis on these kidney diseases have been demonstrated in animal models (Lloyd et al. 1997; Kitagawa et al. 2004; Kang et al. 2010; Sayyed et al. 2011). A number of CCL2/CCR2 axis inhibitors, such as CCX140-B and emapticap pegol, have also been developed, and have demonstrated a degree of clinical benefit for diabetic kidney disease in humans (de Zeeuw et al. 2015; Menne et al. 2017). Inhibition of the CCL2/CCR2 axis is thus suggested to be an effective approach for managing CKD.

The activity of CCL2 is stabilized by the N-terminal pyroglutamate (pE-) residue in vivo, which is formed by the enzymatic activity of glutaminyl cyclase (QC) and isoQC (Cynis et al. 2008). When this conversion of CCL2 is prevented by QC/isoQC inhibitors, CCL2 becomes unstable and is promptly degraded in vivo, leading to a reduced chemoattractive effect for CCR2-expressing cells (Cynis et al. 2011; Ling and Luster 2011; Chen et al. 2012). We therefore hypothesized that inhibiting the QC/isoQC activity would trigger the degradation of CCL2 in vivo and inhibit monocyte and macrophage infiltration and inflammation in the kidney, leading to beneficial effects against CKD.

Here, we evaluated the effects of chronic treatment with PQ529, a potent QC/isoQC inhibitor with a Ki-value (pH 8) for human/murine QC of 38/27 nM and for human/murine iso QC of 4/2 nM (Cynis et al. 2011), on kidney inflammation and CKD conditions in glomerulonephritis model rats. In addition, we evaluated the utility of blood and urine CCL2 levels as pharmacology markers (Morgan et al. 2012) of QC/isoQC inhibitors to predict efficacy in CKD treatment.

## Materials and methods

### QC/isoQC inhibitor

1-(1*H*-Benzimidazol-6-yl)-5-(4-propoxyphenyl)imidazolidine-2,4-dione (PQ529; Probiodrug, Halle, Germany) [Cynis H. 2011] was synthesized at Astellas Pharma Inc. (Ibaraki, Japan).

### Ethics statement

Animals were housed under controlled temperature, humidity, and light (12-h light-dark cycle) conditions and provided a standard commercial diet and water ad libitum. Animals were handled in accordance with the *Guide for the Care and Use of Laboratory Animals*, and all procedures were approved by the Animal Ethics Committee of Astellas Pharma Inc.

### Pharmacokinetics

After oral administration of PQ529 (30 and 100 mg/kg) to nonfasted male Wistar rats, blood (0.2 mL) was drawn via jugular vein cannulation at 0.5, 1, 2, 4, and 6 h and centrifuged to obtain the plasma fraction. The test compound in plasma samples was extracted by deproteination with acetonitrile and then analyzed by liquid chromatography with tandem mass spectrometry (LC-MS/MS).

### Antibody to rat pE-CCL2

Genes of anti-rat pE-CCL2 antibody heavy chain (see Supplementary Fig. S1) and anti-rat pE-CCL2 antibody light chain (see Supplementary Fig. S2) were synthesized (Life Technologies Japan, Tokyo, Japan) and subcloned into pEE6.4 vector and pEE12.4 vector (The GS Gene Expression System™; Lonza, Basel, Switzerland), respectively. Both vectors (0.3 mg for each) were mixed and incubated with 1.2 mL of 293fectin™ Transfection Reagent (Thermo Fisher Scientific, Waltham, MA, USA) in 20 mL of OPTIMEM (Thermo Fisher Scientific) at room temperature (RT) for 30 min. They were then applied to FreeStyleTM 293-F cells (Thermo Fisher Scientific) followed by incubation at 37 °C and 8% CO_2_ for 5 days. The supernatant of the Freestyle 293-F cells was collected and then applied to Protein G Sepharose 4 Fast Flow antibody purification resin (GE Healthcare, Chicago, IL, USA) to purify anti-pE-CCL2 antibody.

### Pharmacodynamics

After 10-min oral administration of PQ529 (30 and 100 mg/kg) to nonfasted male Wistar rats, lipopolysaccharide (LPS; from *Escherichia coli* 055:B5, 0.5 mg/kg, 3,000,000 endotoxin units/mg; Sigma-Aldrich, St. Louis, MO, USA) was injected intraperitoneally. Blood was drawn from the abdominal vena cava using a 19- to 23-gauge needle under isoflurane anesthesia at 0 (prePQ529 treatment), 1, 2, 3, and 5 h and centrifuged at approximately 15,000*g* to measure the levels of plasma pE-CCL2. Heparin was used as the anti-coagulation reagent. Plasma pE-CCL2 levels were measured by enzyme-linked immunosorbent assay (ELISA). Antirat pE-CCL2 antibody binding to ELISA plates (White 384-Well MaxiSorp™ Plates; Thermo Fisher Scientific) was performed in phosphate-buffered saline (PBS) at 4 °C overnight, followed by blocking with 4% BSA and 0.1% Tween 20 in PBS at room temperature for 30 min. After washing (3 times with 0.1% Tween 20 in PBS), plasma samples or standard rat CCL2 (PeproTech, Rocky Hill, NJ, USA) were added and incubated for 2 h at RT, followed by washing (3 times with 0.1% Tween 20 in PBS). Biotin-conjugated anti2C9 antibody diluted in Can Get Signal Solution 1 (TOYOBO, Osaka, Japan) was added and incubated for 2 h at RT, followed by washing. Avidin-conjugated HRP (Thermo Fisher Scientific) diluted in Can Get Signal Solution 2 (TOYOBO) was then added and incubated for 1 to 1.5 h at RT, followed by washing. Finally, SuperSignal™ ELISA Femto Substrate (Thermo Fisher Scientific) was added, and the signal intensity and background of each well was read out and recorded with Envision (PerkinElmer, Waltham, MA, USA).

### Rat model of anti-glomerular basement membrane glomerulonephritis

Male 6-week-old WKY rats were purchased from Charles River Japan (Charles River Laboratories Japan, Inc., Kanagawa, Japan). Rabbit anti-glomerular basement membrane serum (IBL, Fujioka, Japan) was intravenously administered to 6-week-old rats (*N* = 30). Age-matched rats were used as a normal group (*N* = 10). Spontaneously voided urine was collected for 24 h from animals in metabolic cages, and blood samples were taken at weeks 0 (− 1 day of anti-GBM serum injection), 1, 2, and 3. Urinary protein concentrations were measured using a protein assay reagent (Bio-Rad Laboratories, Inc., Hercules, CA, USA). Plasma and urine creatinine and blood urea nitrogen (BUN) levels were measured using a Hitachi 7180 automatic analyzer (Hitachi High-Technologies Corporation, Tokyo, Japan). Serum and urine total CCL2 were measured using an ELISA kit (DAKO, Inc., Carpinteria, CA, USA). Urine KIM-1, β2 microglobulin, and clusterin were measured using WideScreen™ Rat Kidney Toxicity Panel 1 and 2 (Merck, Kenilworth, NJ, USA).

Anti-GBM serum–injected rats were grouped such that urinary protein excretion, plasma creatinine and BUN levels, urine volume, and body weight were uniform among the groups. Group compositions were as follows: (1) normal rats (*N* = 10), anti-GBM serum injected rats treated with (2) vehicle (*N* = 10), (3) PQ529 (30 mg/kg, *N* = 10), and (4) PQ529 (100 mg/kg, *N* = 10). PQ529 was orally administered to the rats twice a day for 3 weeks. At weeks 0, 1, 2, and 3, the serum CCL2 and creatinine, BUN, 24-h urinary protein excretion, urine creatinine, KIM-1, β2 microglobulin, and clusterin levels were measured. After 24-h urinary collection at week 3, blood samples were collected from the abdominal vena cava under isoflurane anesthesia, and a kidney was isolated from each rat and weighed.

### Sample processing

Urine samples collected over 24 h in metabolic cages were centrifuged at 3000–6000*g*, and the supernatant was used to measure urinary protein, creatinine, and other parameters. Blood samples were collected under isoflurane anesthesia from the retroorbital venousplexus (at weeks 0, 1, and 2) using heparin-coated glass capillaries (Terumo Corporation, Tokyo, Japan) or the abdominal vena cava (at week 3) with 19- to 23-gauge needles and centrifuged at approximately 15,000*g* to measure the levels of serum or plasma creatinine, CCL2, and other substances in the supernatant. Heparin was used as the anti-coagulation reagent.

The kidneys were weighed after extraction from the body; in one of the kidneys, the upper half of the renal tissue was immersed in 10% neutral-buffered formalin for histological evaluation, and the cortex of the remaining half was frozen in liquid nitrogen and stored at − 80 °C until processing for mRNA quantification. The other kidney was lysed with Pierce™ IP Lysis Buffer (Thermo Fischer Scientific) containing 1× of Halt™ Protease Inhibitor Cocktail, EDTA-free (Thermo Fischer Scientific) to measure kidney CCL2 using an ELISA Kit (DAKO, Inc.) and kidney IFN-γ using a rat IFN-gamma Quantikine ELISA Kit (R&D systems, Minneapolis, MN, USA).

### Quantification of renal RNA

Total renal RNA was extracted using an RNeasy Mini Kit (QIAGEN, Hilden, Germany) in accordance with the manufacturer’s instructions. Complementary DNA was synthesized using a SuperScript™ VILO™ cDNA Synthesis Kit (Thermo Fischer Scientific). Real-time polymerase chain reaction (PCR) was used to quantify the CD68 and CCL2 gene expression with the following primers: 5’-GAACCCGAACAAAACCAAGGT-3′ and 5’-AGCTGTCCGTAAGGGAATGAGA-3′ for CD68 and 5’-CAGCACCTTTGAATGTGAACTTG-3′ and 5’-TGCTTGAGGTGGTTGTGGAA-3′ for CCL2. Reactions were performed using SYBR Green with an ABI PRISM 7900 Sequence Detection System (Applied Biosystems, Foster City, CA, USA). Data were normalized to endogenous hypoxanthine-guanine phosphoribosyltransferase (HPRT) or β-actin mRNA control.

### Histology

Specimen preparation and histopathological examinations were performed at the Drug Safety Research Laboratories of Astellas Pharma Inc. Renal tissues fixed in 10% neutral-buffered formalin were embedded in paraffin, sectioned at 3 μm, and stained with hematoxylin and eosin (H&E) and periodic acid-Schiff (PAS). Mononuclear cell infiltration into kidney was quantified as previously described (Huan et al. 2014). The total number of interstitial cell clusters, the size of each cluster, and the total cumulative size of the clusters in randomly selected, nonoverlapping 10 visual field per H&E-stained kidney section were determined using Aperio ImageScope version 12.3.3.5048 image analysis software (Leica Biosystems, Wetzlar, Germany). To determine the degree of glomerulosclerosis, a semiquantitative score was obtained from PAS-stained sections by multiplying the grades for segmental and global sclerosis (grade 0: no damage, 1: segmental sclerosis in < 50%, 2: segmental sclerosis in ≥ 50%, 3: global sclerosis in < 25%, 4: global sclerosis in ≥ 25% of the glomeruli in the visual field). To determine the degree of tubulointerstitial damage, a semiquantitative score was obtained from H&E-stained sections by multiplying the grades for dilation of the renal tubules, thickening of the basement membrane, urinary cast, basophilic changes in the tubular epithelium, and hyaline droplet deposition (grade 0: no damage, 1: damage in 25%, 2: damage in half, 3: damage in 75%, 4: damage in the whole area of the renal cortex in the visual field).

### Statistical analyses

The results are expressed as the mean ± standard deviation (S.D.). Significant differences between two groups were assessed using Student’s *t* test, while those among multiple groups were assessed using a one-way analysis of variance followed by Dunnett’s multiple comparisons test as a post hoc test. Correlations were analyzed using Pearson’s rank correlation. Histopathological scores were compared using a Mann–Whitney test to analyze differences between two groups, while a nonparametric Kruskal–Wallis analysis of variance followed by Dunn’s multiple comparisons test was used for comparisons among multiple groups. A value of *p* < 0.05 was considered significant. Statistical and data analyses were conducted using GraphPad Prism 8.0.2 (GraphPad Software, San Diego, CA, USA).

## Results

### Pharmacokinetics and pharmacodynamics of PQ529

**Fig. 1:**
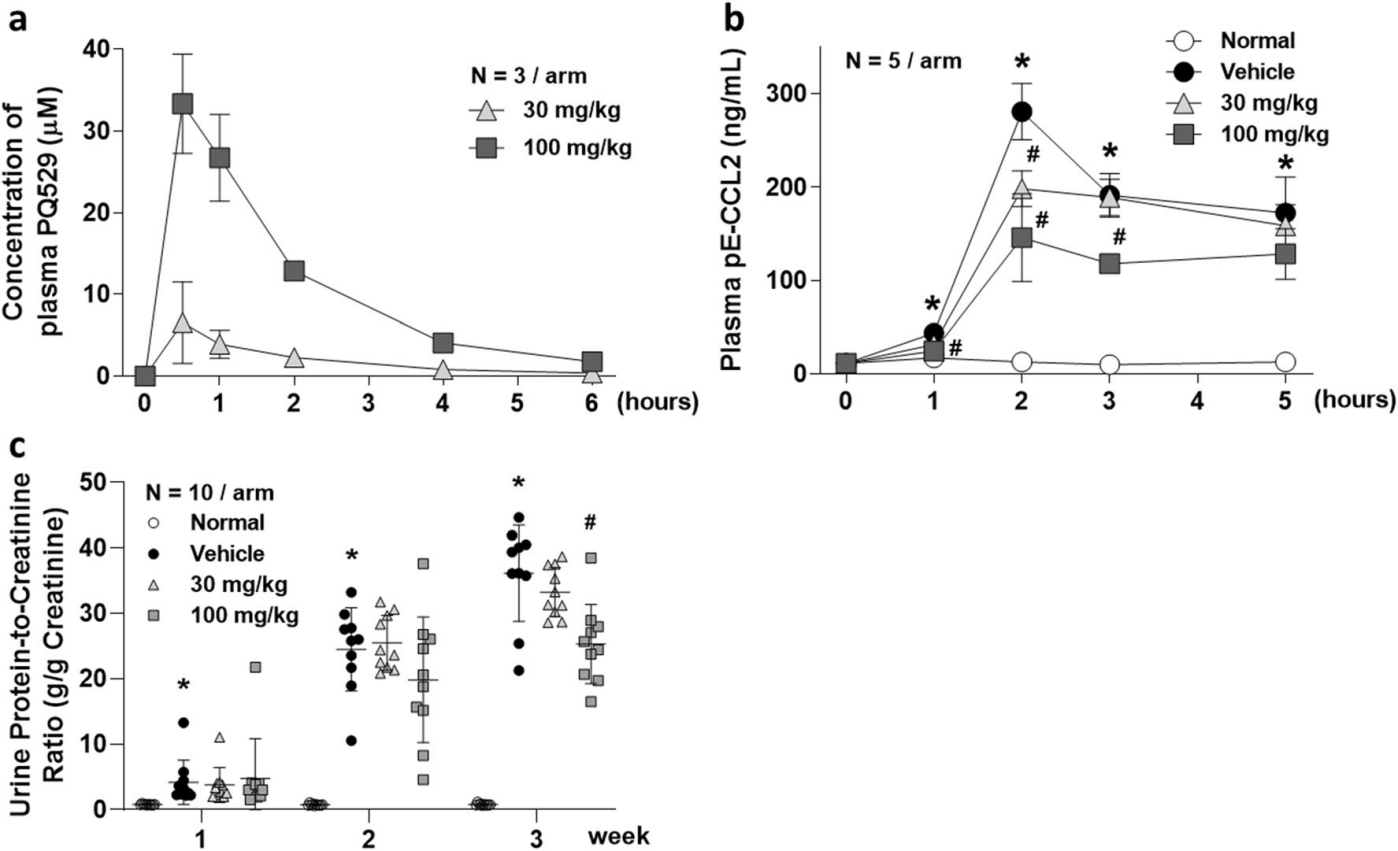
Pharmacokinetics and pharmacodynamics of PQ529 in Wistar and WKY rats and effect of repeated administration of PQ529 on the urinary protein excretion in anti-GBM-induced glomerulonephritis rats. **a** Time course of changes in the plasma concentration of the unchanged PQ529 (30 and 100 mg/kg) in male Wistar rats. **b** Time course of changes in the plasma concentration of pE-CCL2 after the administration of PQ529 (30 and 100 mg/kg) to WKY rats. **c** PQ529 was orally administered to anti-GBM-induced glomerulonephritis rats twice daily for 3 weeks. Data are expressed as the mean ± S.D. for **a** three Wistar rats and **b** five WKY rats per sampling point and **c** 10 WKY rats per group. The results are expressed as the mean ± standard deviation (S.D.). Significant differences between two groups were assessed using Student’s *t* test, while those among multiple groups were assessed using a one-way analysis of variance followed by Dunnett’s multiple comparisons test as a post hoc test. *p < 0.05 vs. normal group, #*p* < 0.05 vs. vehicle group

After oral administration of PQ529 (30 and 100 mg/kg) to Wistar rats, the plasma concentration of unchanged drug peaked at 30 min (6.5 and 33 μM of plasma concentrations at 30 and 100 mg/kg of PQ529, respectively) and then gradually decreased (Fig. 1a), with a terminal half-life of 0.5–1.0 h. We confirmed that the ratio of the free portion of this compound was 12%, suggesting that about 784 and 4000 nM of protein-unbound free PQ529 was present in the blood at 0.5 h after dosing at 30 and 100 mg/kg, respectively.

We also found the IC_50_ of the inhibitory effects of PQ529 on human, mouse, and rat isoQC enzymatic activity to be 37, 43, and 20 nM, respectively. In addition, we found the IC_50_ of the QC/isoQC activity in mouse Raw264.7 and human THP-1 cells to be 630 and 1500 nM, respectively (data not shown). In our pharmacodynamic study, 30 and 100-mg/kg PQ529 were confirmed to inhibit the LPS-induced production of pE-CCL2 by approximately 30% and 50%, respectively, at 2 h after the injection of LPS (0.5 mg/kg) (Fig. 1b).

### Effects of repeated administration of PQ529 on kidney function and amount of CCL2 in plasma, urine, and kidney of glomerulonephritis rats

**Fig. 2:**
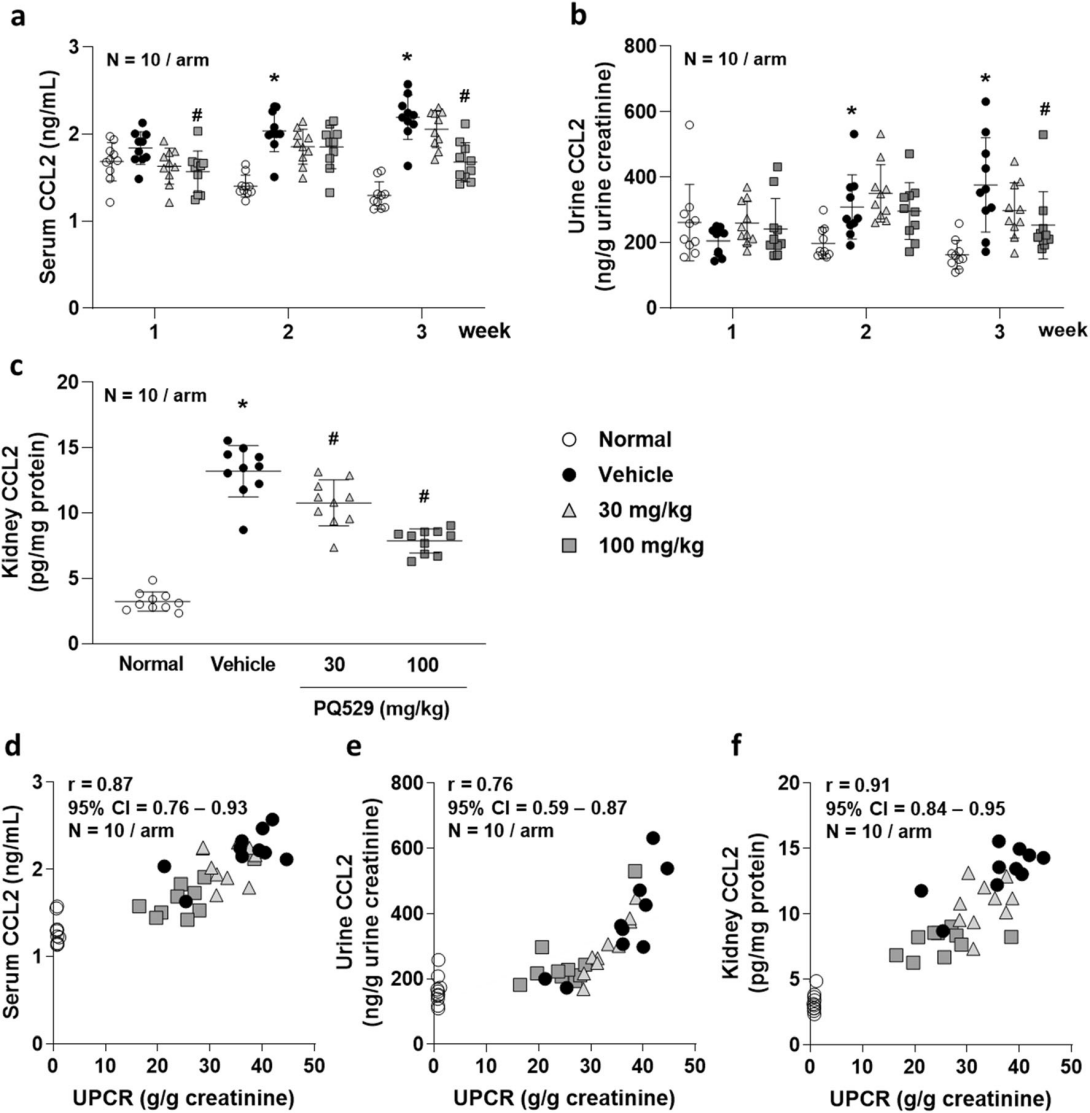
Effect of repeated administration of PQ529 on **a** serum, **b** urine, and **c** kidney CCL2 and correlation between **d** serum, **e** urine, and **f** kidney CCL2 and urine protein-to-creatinine ratio in anti-GBM-induced glomerulonephritis rats. PQ529 was orally administered to anti-GBM-induced glomerulonephritis rats twice daily for 3 weeks. Data are expressed as the mean ± S.D. for 10 animals per group. The results are expressed as the mean ± standard deviation (S.D.). Significant differences between two groups were assessed using Student’s *t* test, while those among multiple groups were assessed using a one-way analysis of variance followed by Dunnett’s multiple comparisons test as a post hoc test. Correlations were analyzed using Pearson’s rank correlation. *p < 0.05 vs. normal group, #*p* < 0.05 vs. vehicle group

No deaths occurred in any group during the drug administration period. Compared with normal rats, the anti-GBM-induced glomerulonephritis rats showed progressive elevation of urinary protein excretion and BUN. Repeated administration of PQ529 (30 and 100 mg/kg) dose-dependently improved the elevated urinary protein excretion and BUN at week 3 (Fig. 1c and Table 1). In addition, total CCL2 concentration in serum and urine was significantly increased in the glomerulonephritis rats beyond 2 weeks after the injection of anti-GBM serum. CCL2 expression in kidney was also significantly increased at necropsy in the glomerulonephritis rats. Repeated administration of PQ529 (30 and 100 mg/kg) suppressed the elevation of CCL2 levels in serum, urine, and kidney in a dose-dependent manner (Fig. 2 a, b, and c) after 3 weeks of PQ529 treatment.

**Table 1:** Effect of repeated administration of PQ529 on general parameters in glomerulonephritis rats

Index	Normal Vehicle	Vehicle	Glomerulonephritis 30 mg/kg	100 mg/kg
Body weight (g)	265 ± 17	252 ± 8*	257 ± 9	257 ± 11
Food intake (g/day)	17.1	16.1	16.7	17.5
Creatinine clearance (mL/day/100 g body weight)	1397 ± 98	893 ± 131*	854 ± 190	1041 ± 154
Blood urea nitrogen (mg/dL)	13.2 ± 1.5	18.0 ± 4.6*	16.4 ± 2.8	13.6 ± 2.5^#^

*p < 0.05 vs. normal group using Student’s *t* test

#p < 0.05 vs. glomerulonephritis vehicle group using Dunnett’s multiple comparison test

PQ529 was orally administered to glomerulonephritis rats twice daily for 3 weeks. The values are the mean ± standard deviation for 10 animals per group

Correlations among the urinary protein excretion and serum (*r* = 0.87, 95% confidence interval [CI] = 0.76–0.93, *p* < 0.01), urine (*r* = 0.76, 95% CI = 0.59–0.87, *p* < 0.01), and kidney (*r* = 0.91, 95% CI = 0.84–0.95, *p* < 0.01) CCL2 levels were confirmed in all tested WKY rats (Fig. 2 d, e, and f).

### Effects of repeated administration of PQ529 on mononuclear cell infiltration into kidney and glomerulosclerosis and tubulointerstitial injury in glomerulonephritis rats

**Fig. 3:**
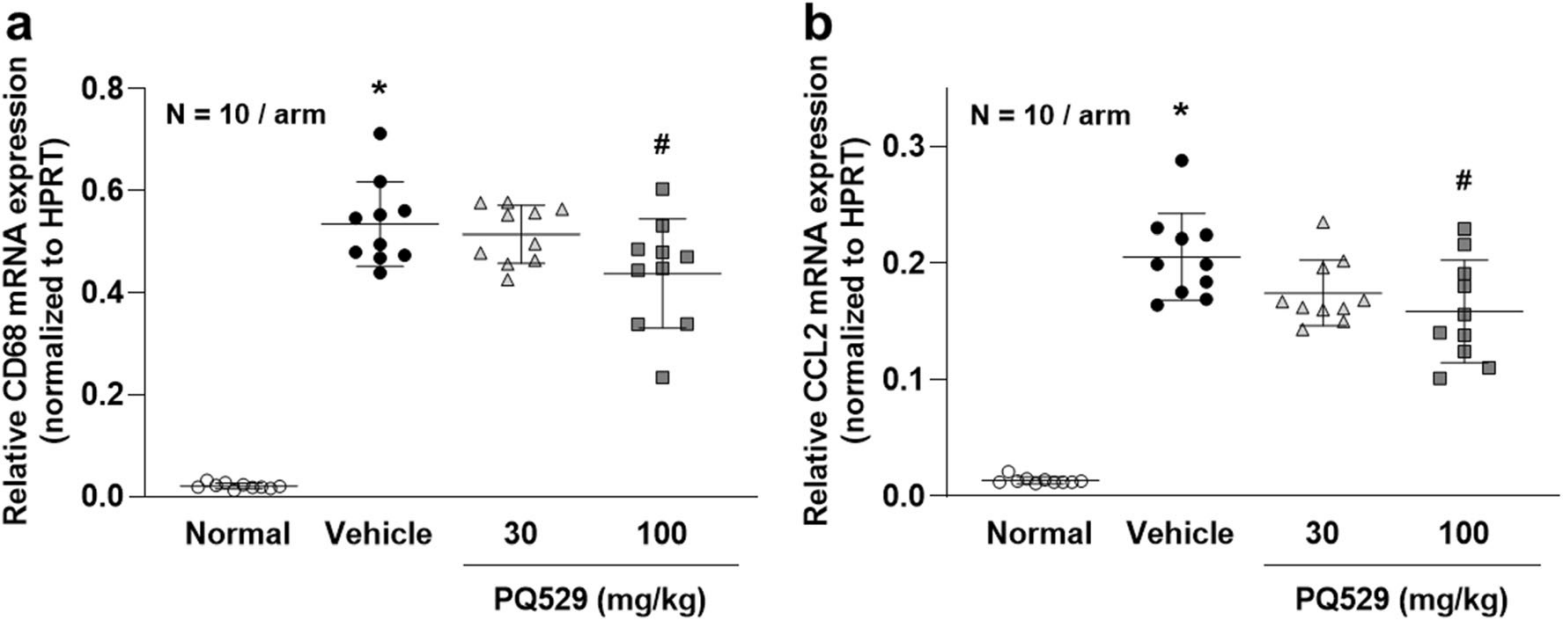
Effect of repeated administration of PQ529 on renal **a** CD68 and **b** CCL2 expression in anti-GBM-induced glomerulonephritis rats. PQ529 was orally administered to anti-GBM-induced glomerulonephritis rats twice daily for 3 weeks. Data are expressed as the mean ± S.D. for 10 animals per group. The results are expressed as the mean ± standard deviation (S.D.). Significant differences between two groups were assessed using Student’s *t* test, while those among multiple groups were assessed using a one-way analysis of variance followed by Dunnett’s multiple comparisons test as a post hoc test. *p < 0.05 vs. normal group, #*p* < 0.05 vs. vehicle group

**Fig. 4:**
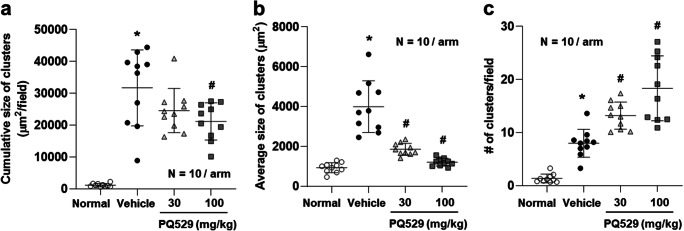
Effect of repeated administration of PQ529 on mononuclear cell infiltration in glomerulonephritis rats. PQ529 was orally administered to glomerulonephritis rats twice daily for 3 weeks. Data are expressed as the mean ± S.D. for 10 animals per group. Significant differences between two groups were assessed using Student’s *t* test, while those among multiple groups were assessed using a one-way analysis of variance followed by Dunnett’s multiple comparisons test as a post hoc test. *p < 0.05 vs. normal group, #*p* < 0.05 vs. vehicle group

**Fig. 5:**
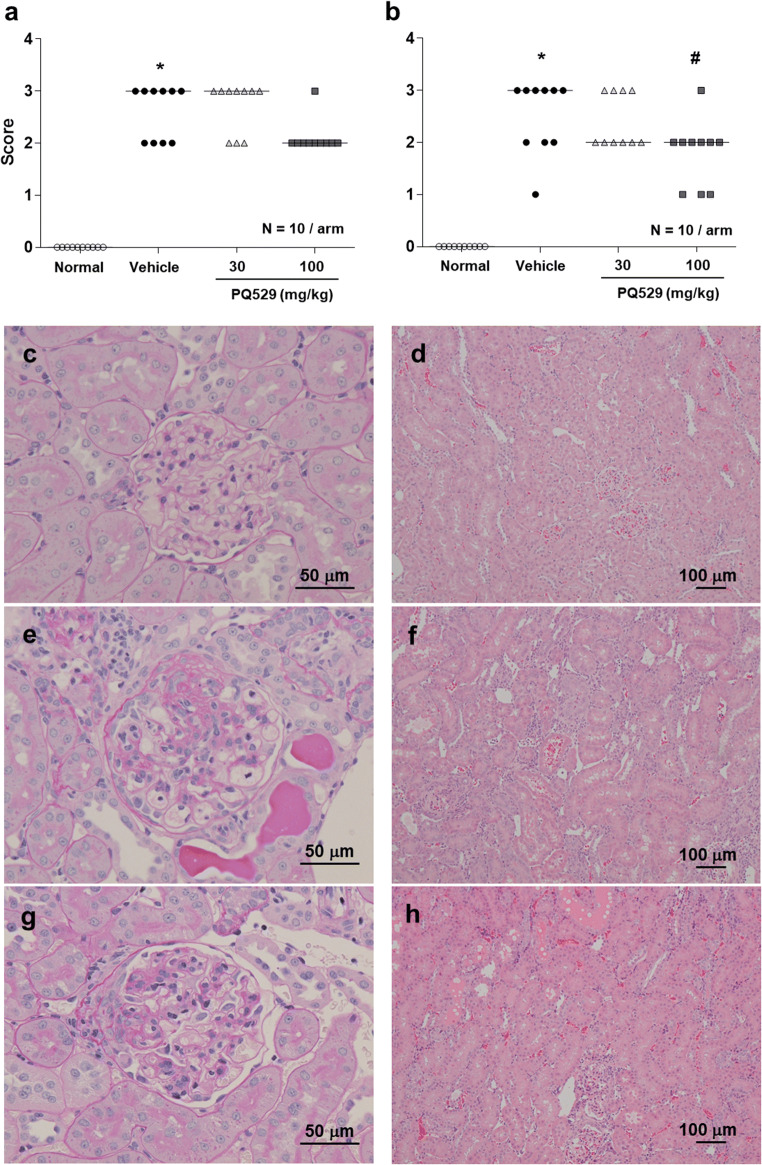
Effect of repeated administration of PQ529 on **a** glomerulosclerosis and **b** tubulointerstitial damage in glomerulonephritis rats and representative light micrographs of renal tissues obtained from **c**, **d** normal (semiquantitative scores of glomerulosclerosis, tubulointerstitial damage: 0, 0), **e**, **f** glomerulonephritis (scores: 3, 3), and **g**, **h** PQ529 (100 mg/kg)-treated rats (scores: 2, 1). Magnification: left, × 40 (PAS stain); right, × 10 (H&E stain). PQ529 was orally administered to glomerulonephritis rats twice daily for 3 weeks. Data are expressed as the median for 10 animals per group. Histopathological nonparametric scores were compared using a Mann–Whitney test to analyze differences between two groups, while a nonparametric Kruskal–Wallis analysis of variance followed by Dunn’s multiple comparisons test was used for comparisons among multiple groups. *p < 0.05 vs. normal group, #*p* < 0.05 vs. vehicle group

In the glomerulonephritis rats, the mRNA levels of CD68 and CCL2 in the kidney cortex were significantly increased at necropsy and significantly suppressed by repeated administration of PQ529 in a dose-dependent manner (Fig. 3a and b). Histological analyses of the glomerulonephritis rat kidney showed increased mononuclear infiltration into the interstitium, which was suppressed by the repeated administration of PQ529 (Fig. 4 a, b, and c). In addition, PQ529 improved the scores of kidney injury, including glomerulosclerosis and tubulointerstitial injury, at 100 mg/kg (Fig. 5 a and b).

**Fig. 6:**
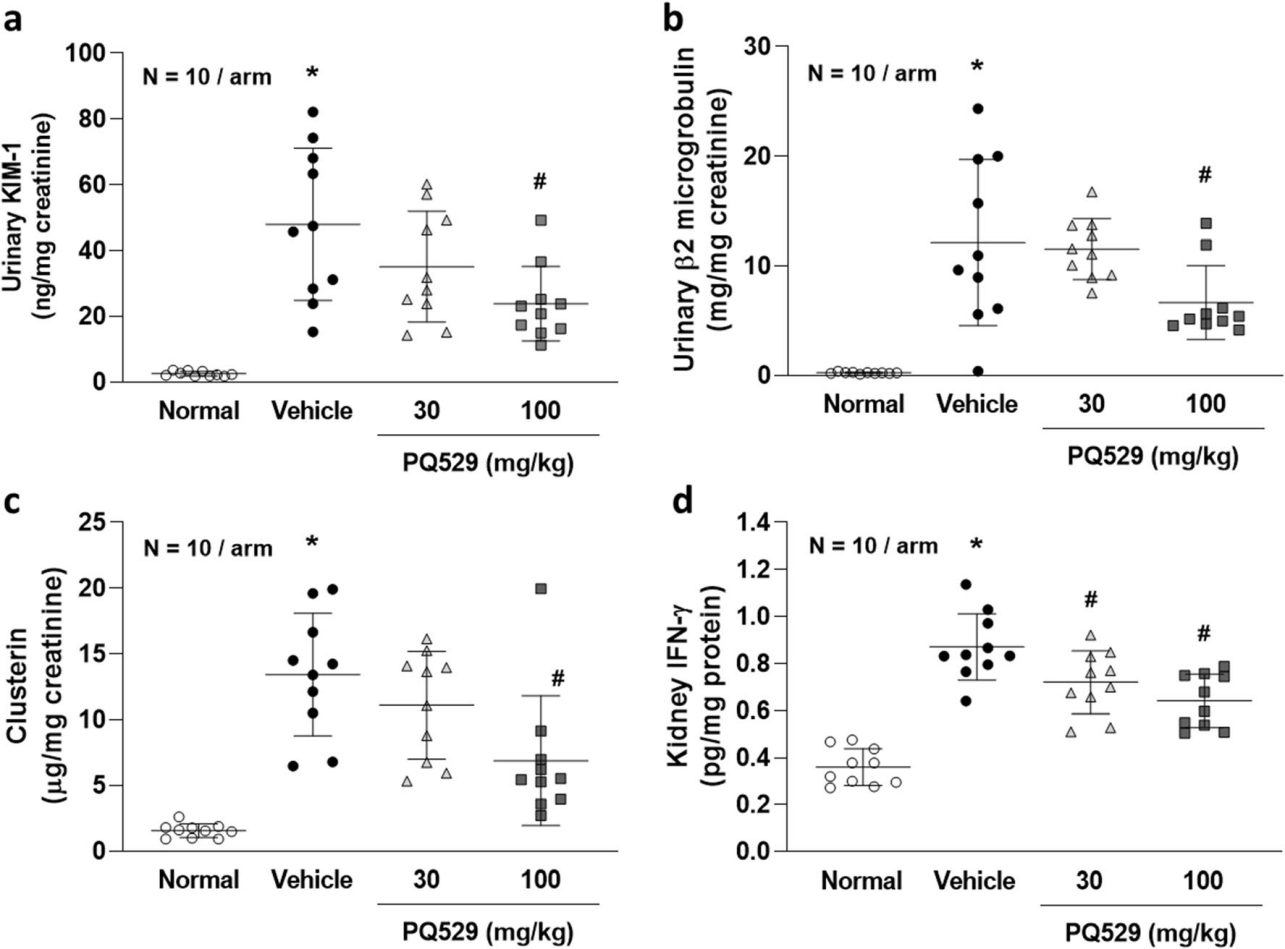
Effect of repeated administration of PQ529 on the urinary **a** KIM-1, **b** β2 microglobulin, and **c** clusterin levels and **d** kidney IFN-γ levels in anti-GBM-induced glomerulonephritis rats. PQ529 was orally administered to anti-GBM-induced glomerulonephritis rats twice daily for 3 weeks. Data are expressed as the mean ± S.D. for 10 animals per group. Significant differences between two groups were assessed using Student’s *t* test, while those among multiple groups were assessed using a one-way analysis of variance followed by Dunnett’s multiple comparisons test as a post hoc test. *p < 0.05 vs. normal group, #*p* < 0.05 vs. vehicle group

Compared with the normal rats, the anti-GBM mAb–induced glomerulonephritis rats showed progressive elevation of the kidney injury molecule-1 (KIM-1), β2 microglobulin, and clusterin levels in the urine as well as IFN-γ expression in the kidneys. Repeated administration of PQ529 dose-dependently improved the elevated levels of KIM-1, β2 microglobulin, and clusterin and the IFN-γ expresion in the kidneys, and these effects were significant at 100 mg/kg (Fig. 6 a, b, c, and d).

## Discussion

The final common pathway in CKD (Hodgkins and Schnaper 2012) is well known to include a correlation between macrophage infiltration and kidney fibrosis (Yonemoto et al. 2006). The CCL2/CCR2 axis contributes to the mononuclear cell infiltration and inflammation in the kidney (Tesch et al. 1999; Giunti et al. 2010; Tampe and Zeisberg 2014; Haller et al. 2016; Moreno et al. 2018), and blockage of this axis exerts beneficial effects in experimental CKD models (Lloyd et al. 1997; Kitagawa et al. 2004; Kang et al. 2010; Sayyed et al. 2011) and human clinical CKD studies (de Zeeuw et al. 2015; Perez-Gomez et al. 2016; Menne et al. 2017). Further, the urinary CCL2 level reportedly correlates with urinary protein leakage (Banba et al. 2000; Eardley et al. 2006) and is a urinary disease biomarker in CKD (Verhave et al. 2013; Siddiqui et al. 2019). Genetic variants of CCL2 are also correlated with the IgA nephropathy risk in humans (Gao et al. 2016). The present study evaluated the protective effects of the QC/isoQC inhibitor PQ529 on progressive renal dysfunction in anti-GBM serum–induced glomerulonephritis rats, reinforcing the importance of blocking the CCL2/CCR2 axis for the treatment of CKD and suggesting a novel approach to achieving such blockade.

The anti-GBM serum–induced glomerulonephritis rat model is often used to investigate the mechanism of CKD and potential therapeutic approaches to preventing CKD progression with inflammation. In this model, CCL2 expression is immediately increased in the tubulointerstitium and glomeruli after the injection of anti-GBM serum (Tang et al. 1996; Natori et al. 1997). These elevated CCL2 levels in the kidneys are also reportedly involved in the progression of tubulointerstitial fibrosis, glomerulonephritis, and massive proteinuria accompanied by monocyte and macrophage recruitment in the kidneys in rats (Taniguchi et al. 2007). Treatment with anti-CCL2 antibodies and CCR2 inhibitors suppresses urine protein leakage and inflammation in the tubulointerstitium and glomeruli and decreases the migration of macrophages into these tissues in a rat model of glomerulonephritis (Wada and Yokoyama 1996). In the present study, chronic treatment of PQ529 significantly decreased systemic (serum and urine) and local (kidney) CCL2 levels, suggesting that QC/isoQC inhibits the production of pE-modified CCL2 systemically, resulting in the degradation of CCL2 in vivo. Several reports have described the involvement of the CCL2/CCR2 axis in the final common pathway in animal models of diabetic kidney disease, and inhibitors of the CCL2/CCR2 axis were effective in ameliorating CKD conditions in these models (Kang et al. 2010; Sayyed et al. 2011). Additionally, further evaluation using other QC/isoQC inhibitors with better in vitro and in vivo profiles will tell maximum efficacy of inhibition of CCL2/CCR2 axis in the studied glomerulonephritis model, while the present study showed partial inhibitory effects of PQ529 on systemic and local CCL2 levels. Taken together, these reports suggest that QC/isoQC inhibitors are effective against not only glomerulonephritis but also inflammation in diabetic CKD kidneys.

In our present glomerulonephritis model study, we detected CCL2 levels as total CCL2 levels using a purchased ELISA kit that detects epitopes other than pE-N-terminal amino acids. We used this particular approach because while the pE-CCL2 levels were easily detected in the rat PD model, these levels in the rat glomerulonephritis model were below the detection limit of our ELISA system. However, given that total CCL2 levels were decreased after chronic treatment of PQ529, this QC/isoQC inhibitor appears to have been effective in this model as an inhibitor of the CCL2/CCR2 axis.

Several previous reports have suggested that blood CCL2 induces the migration of CCR2-expressing monocytes through the bone marrow to the blood stream, and that CCL2 in the tubulointerstitium induces macrophage infiltration at inflammatory sites in the kidneys in CKD (Sayyed et al. 2011). However, the number of macrophages in the blood was not evaluated in these studies, so whether PQ529 treatment induces the migration of monocytes from the bone marrow to the blood stream in addition to monocyte infiltration into kidneys in CKD should be examined in future studies. We confirmed in the present study that the degree of decrease in CCL2 protein in the kidney was similar to the degree of decrease in the infiltration of mononuclear cells into the kidney, as shown by our comparison of the control and PQ529-treated glomerulonephritis rats. However, the decrease in CD68 level in the kidneys appeared to be less than the outcomes of semiquantitative scoring of infiltration of mononuclear cells. As the histopathological data are evaluated by semiquantitative scores, comparing the degree of efficacy of PQ529 on CD68 levels in the kidney with that on mononuclear cell infiltration into the kidney may not be appropriate, as a gap exists between these two data sets. To quantify this gap, a more detailed evaluation, such as assessment of the time course of macrophage infiltration by immunohistochemistry, will need to be conducted.

We confirmed the decrease in the CCL2 mRNA levels in PQ529-treated WKY rats, which may have been due to a reduction in inflammation in the tubulointerstitium, possibly as a result of reduced macrophage infiltration in the kidneys. We further confirmed reduced levels of tubular injury markers, such as KIM-1, β2 microglobulin, and clusterin, in urine samples; a decreased IFN-γ expression in the kidney; and decreased tubulointerstitial injury on histochemical analyses. We also observed a trend toward decreased glomerulosclerosis injury in the kidneys of PQ529-treated rats. Taken together, these data suggest a relationship between reduced mononuclear cells or macrophages and reduced kidney injury by treatment with PQ529, although the correlation was difficult to assess due to the limitations associated with the semiquantitative analysis of histopathological observations. In addition, it has been reported that several other chemokines such as RANTES, IL-8, or PDGF were also closely involved in the development of glomerulonephritis (Stasikowska and Wągrowska-Danilewicz 2007). However, in this study, we have measured some chemokine and cytokines only; further investigation is necessary to clarify these issues.

In the PQ529-treated glomerulonephritis rats, decreased levels of urinary protein were observed along with reduced inflammation in the kidney. These data underscore the usefulness of inhibitors of the CCL2/CCR2 axis. Further, following treatment with PQ529, both serum and urinary total CCL2 levels were significantly decreased in a PQ529-dose dependent manner, with statistically significant correlations detected between urine protein levels and the serum and urine CCL2 levels. In recent years, the importance of confirming proof of pharmacology in the early stage of drug development has been reported (Morgan et al. 2012). Given the present findings, both the serum and urine CCL2 levels are considered biomarkers of the proof of pharmacology for CKD, strengthening this QC/isoQC inhibition approach for managing CKD over other inhibitors of CCL2/CCR2 that lack such a biomarker.

Recently, phase 2 studies of certain inhibitors of the CCL2/CCR2 axis, such as CCX140-B and emapticap pegol, have been conducted in diabetic kidney disease patients. Neither of these studies showed a statistically significant decrease in urine protein compared with placebo in chronic treatment, but a tendency toward improvement was noted. In the phase 2 study of CCX140-B, a low dose (5 mg) was found to be more effective than a high dose (10 mg), with compensatory increased blood CCL2 levels observed in the high-dose cohort (de Zeeuw et al. 2015). While the reason for this compensatory increase in CCL2 on treatment with CCX140-B remains unclear, we confirmed that CCX140-B itself is able to increase the LPS-induced pE-CCL2 expression in human THP-1 cells, while PQ529 contrarily reduced its production (data not shown). In addition, we confirmed in the present study that chronic treatment with PQ529 decreased CCL2 mRNA levels in inflammatory kidney in this study, suggesting that this compound has no compensatory effects on CCL2 expression in vivo. Given these findings, compensatory increases in CCL2 are not expected to occur during chronic treatment with QC/isoQC inhibitors in humans, a notion supported by the decreased serum, urine, and kidney CCL2 levels in PQ529-treated glomerulonephritis rats. Therefore, monitoring for decreased serum and urine CCL2 levels as a proof of pharmacology biomarker is expected to be useful for estimating effective doses of QC/isoQC inhibitors in humans and confirming whether or not a QC/isoQC inhibitor will exert the expected pharmacological effects in vivo in human clinical trials.

In conclusion, the present findings suggest that the CCL2/CCR2 axis plays an important role in glomerulonephritis in CKD and that the QC/isoQC inhibitor PQ529 suppresses this axis by inhibiting the modification of glutamine with a pyroglutamate residue at the CCL2 N-terminal, consequently suppressing the inflammation in the kidney and exerting inhibitory effects on the progression of chronic renal failure in glomerulonephritis rats. These data suggest that chronic treatment with QC/isoQC inhibitors is a novel and effective approach to managing glomerulonephritis and CKD.

## Supplementary Information

ESM 1 (XLSX 91 kb)

ESM 2 (DOCX 24 kb)
