# Supplementary material for: Chronic treatment with the (iso-)glutaminyl cyclase inhibitor PQ529 is a novel and effective approach for glomerulonephritis in chronic kidney disease
Source: Naunyn Schmiedebergs Arch Pharmacol. 2021 Mar 29;394(4):751–61. doi: 10.1007/s00210-020-02013-x (PMC8007495; doi:10.1007/s00210-020-02013-x)
Supplement: Supplementary file 2 — (DOCX 24 kb) [file 210_2020_2013_MOESM2_ESM.docx]

**Supplementary Figures**

## Chronic treatment with the (iso-)glutaminyl cyclase inhibitor PQ529 is a novel and effective approach for glomerulonephritis in chronic kidney disease

Naotoshi Kanemitsu^1^, Fumiko Kiyonaga^2^, Kazuhiko Mizukami^3^, Kyoichi Maeno^3^, Takashi Nishikubo^4^, Hiroyuki Yoshida^3^, Hiroyuki Ito^3^,

**Figure S1. DNA sequence of anti-rat pE-CCL2 antibody heavy chain**

(5’-)

AAGCTTGCCGCCACCATGGAATGGTCCTGGGTGTTCCTGTTCTTCCTGAGCGTGACCACCGGCGTGCACAGCGAAGTGCAGCTGCAGCAGTCTGGCCCCGAGCTCGTGAAACCTGGCGCCTCCGTGAAGATGAGCTGCAAGGCCAGCGGCTACACCTTCACCGACTACTACATGGACTGGGTCAAGCAGAGCCACGGCGAGAGCTTCGAGTGCATCGGCAGAGTGAACCCCTACAACGGCGGCACCAGCTACAACCAGAAGTTCAAGGGCAAGGCCACCCTGACCGTGGACAAGAGCAGCAGCACCGCCTACATGGAACTGAACAGCCTGACCAGCGAGGACAGCGCCGTGTACTACTGTGCCAGACTGGGCAGCAGCTACAGATGGGGCCAGGGCACAACCCTGACAGTGTCCAGCGCCAAGACCACCCCCCCTAGCGTGTACCCTCTGGCCCCTGGATCTGCCGCCCAGACCAACAGCATGGTCACCCTGGGCTGCCTCGTGAAGGGCTACTTCCCTGAGCCTGTGACCGTGACCTGGAACAGCGGCTCTCTGAGCAGCGGAGTGCACACCTTCCCTGCCGTGCTGGAAAGCGACCTGTACACCCTGAGCAGCTCCGTGACCGTGCCCAGCAGCCCTAGACCTAGCGAGACAGTGACCTGCAACGTGGCCCACCCTGCCAGCAGCACAAAGGTGGACAAAAAGATCGTGCCCAGAGACTGCGGCTGCAAGCCCTGCATCTGCACCGTGCCTGAGGTGTCCTCCGTGTTCATCTTCCCACCCAAGCCCAAGGACGTGCTGACCATCACCCTGACACCCAAAGTGACCTGTGTGGTGGTGGACATCAGCAAGGACGACCCCGAGGTGCAGTTCAGTTGGTTCGTGGACGACGTGGAAGTGCACACAGCCCAGACCCAGCCCAGAGAGGAACAGTTCAACAGCACCTTCAGAAGCGTGTCCGAGCTGCCCATCATGCACCAGGACTGGCTGAACGGCAAAGAGTTCAAGTGTAGAGTGAACAGCGCCGCCTTCCCAGCCCCCATCGAAAAGACCATCTCCAAGACCAAGGGCAGACCCAAGGCCCCCCAGGTGTACACAATCCCCCCACCCAAAGAACAGATGGCCAAGGACAAGGTGTCCCTGACCTGCATGATCACCGATTTCTTCCCAGAGGACATCACCGTGGAATGGCAGTGGAACGGCCAGCCCGCCGAGAACTACAAGAACACACAGCCTATCATGAACACCAACGGCAGCTACTTCGTGTACAGCAAGCTGAACGTGCAGAAGTCCAACTGGGAGGCCGGCAACACCTTTACCTGCAGCGTGCTGCACGAGGGCCTGCACAATCACCACACCGAGAAGTCCCTGTCCCACAGCCCCGGCAAATGAGAATTC (-3’)

**Figure S2. DNA sequence of anti-rat pE-CCL2 antibody light chain**

(5’-) AAGCTTGCCGCCACCATGAGCGTGCCAACACAGGTGCTGGGACTGCTGCTGCTGTGGCTGACCGACGCCAGATGCGACGTCGTGATGACCCAGACCCCTCTGAGCCTGAGCGTGACCATCGGCCAGCCTGCCAGCATCAGCTGCAAGAGCAGCCAGAGCCTGCTGGACAGCGCCGGCAAGACATACCTGAGCTGGCTGCTGCAGAGGCCTGGCCAGAGCCCCAAGAGACTGATCTACCTGGTGTCCAAGCTGGACTCCGGCGTGCCCGACAGATTCACAGGATCTGGCAGCGGCACCGACTTCACCCTGAAGATCAGCAGAGTGGAAGCCGAGGACCTGGGCGTGTACTACTGTTGGCAGGGCACACACTTCCCTTGGACCTTCGGCGGAGGCACAAAGCTGGAAATCAAGAGAGCCGACGCCGCTCCCACCGTGTCCATCTTCCCACCTAGCAGCGAGCAGCTGACCTCTGGCGGAGCTAGCGTCGTGTGCTTCCTGAACAACTTCTACCCCAAGGACATCAACGTGAAGTGGAAGATCGACGGCAGCGAGAGACAGAACGGCGTGCTGAACAGCTGGACCGACCAGGACAGCAAGGACTCCACCTACAGCATGAGCAGCACCCTGACCCTGACCAAGGACGAGTACGAGAGACACAACAGCTACACATGCGAGGCCACCCACAAGACCAGCACCAGCCCCATCGTGAAGTCCTTCAACAGAAACGAGTGCTGAGAATTC (-3’)
